# Barriers and facilitators in the implementation of youth and young adult models of mental health care

**DOI:** 10.1111/eip.13555

**Published:** 2024-05-20

**Authors:** Joanna Reed, Lucy Hunn, Tiffany Smith, Robyn Bosworth, Brioney Gee, Clio Berry, Timothy Clarke

**Affiliations:** ^1^ Department of Clinical Psychology and Psychological Therapies University of East Anglia Norwich UK; ^2^ Norfolk and Suffolk NHS Foundation Trust Norwich UK; ^3^ Department of Primary Care and Public Health Brighton and Sussex Medical School Brighton UK

**Keywords:** adolescent mental health, implementation, qualitative, young adult mental health, youth models of mental health care

## Abstract

**Aim:**

It is increasingly recognised that traditional models of mental health (MH) care, with a service transition at age 18 years, may not reflect best practice. The literature supports a move towards youth and young adult focused models of MH care, for young people up to the age of 25, which specifically cater to the unique psychosocial and developmental needs of this population. This service evaluation aimed to explore the facilitators and barriers to the implementation of youth models of MH care across England (UK).

**Methods:**

Six services participated in separate focus groups pertaining to their experience of implementing youth models of MH care. The interview guide for the focus groups was informed by the Consolidated Framework for Implementation Research (CFIR) and explored barriers and facilitators to implementation and sustainment. The focus groups were recorded, transcribed verbatim and analysed thematically.

**Results:**

Seven key themes relevant to the implementation of youth models of MH care were identified: a clear rationale for doing things differently, for young people by young people, “building those relationships is key”, service identity development, resource and infrastructure, leadership at multiple levels, and valuing and developing staff.

**Conclusions:**

The findings suggest effective communication and leadership, co‐production and cross system collaboration contribute to successful implementation of youth models of MH care. The findings will be of interest to those involved in informing and supporting successful implementation and delivery of youth models of mental health care at local and national levels.

## 1 INTRODUCTION

A recent meta‐analysis estimated that around 60% of mental health difficulties first occur before the age of 25 years (Solmi et al., 2022). Therefore, adolescence and young adulthood is a critical time during which mental health services can intervene to improve long‐term mental health outcomes. Increasing evidence suggests that the traditional structure of National and International Child and Adolescent Mental Health services (CAMHS) supporting those up to 18 years old, and Adult Mental Health Services (AMHS) supporting those 18 and over, does not reflect a best practice approach to youth mental health (Fusar‐Poli, 2019). Young people report that transitions between CAMHS and AMHS can be experienced as disempowering and may exacerbate their mental health difficulties (Street et al., 2018). Furthermore, research has highlighted that young adults have distinct developmental and clinical needs and may be less likely to engage with AMHS (Fusar‐Poli, 2019; Roche et al., 2019).

The International Declaration on Youth Mental Health (Coughlan et al., 2013) has called for a transformation of the way mental health services are delivered. It details a shared vision, principles, and actions for addressing young people's mental health needs in a way that is responsive to developmental processes taking place as a young person transition into adulthood. Additionally, the NHS long‐term plan (NHS England, 2019) emphasises the need for a comprehensive service offer for 0–25‐year‐olds that reaches across mental health services for children, young people and adults. Increasingly, services and systems have begun implementing youth or young adult models of mental health care for 0–25‐year‐olds (these terms are used interchangeably and will henceforth be referred to as youth models) to bridge transitions between CAMHS and AMHS.

Recommendations for key service components and guiding principles of youth models of mental health care, such as co‐production and accessibility of services, have been outlined in the literature (Gossip et al., 2021; Howe et al., 2014; McGorry & Mei, 2020; National Collaboration Centre for Mental Health, 2019). Whilst there is accumulating literature detailing the principles of youth models of mental health care, there has been limited consideration as to the process of translating these into routine care. Proctor et al. (2009) highlights the importance of implementation science research in bridging conceptual knowledge of effective mental health care and what is ultimately provided by services.

The current service evaluation aimed to explore the barriers and facilitators experienced by services in England (UK) when implementing youth‐focused models of mental health care. Awareness of key implementation facilitators and barriers can help systems to develop multi‐faceted plans for implementing innovations in mental health care delivery tailored to their local context (Powell et al., 2012). It is therefore intended that the findings presented will be of interest to those in the process of implementing and sustaining youth models of mental health care, whose actions will hopefully contribute to greater national equity in the availability of youth‐focused service provision.

## 2 METHOD

Six different services in England (see Table S1) participated in separate focus groups between July and August 2022, exploring their experiences of implementing youth models of mental health care. Services were recruited via email through national youth mental health networks and word‐of‐mouth. To be included, services must have implemented an established model of youth mental health care.

A total of 31 professionals, including senior organisational managers, commissioners, service managers, and clinicians, participated, with a minimum of three participants per focus group. The focus groups were conducted by TC, JR and LH using videoconferencing software and lasted approximately 90 min. A minimum of two facilitators were present for each focus group. An interview guide was developed based on the Consolidated Framework for Implementation Research (CFIR; Damschroder et al., 2009; see Table S1). The CFIR framework draws upon extensive empirical findings relevant to implementation science to provide an array of constructs associated with successful implementation, and organises these into five core domains: intervention characteristics, outer setting, inner setting, characteristics of individuals, and process. The framework can be used to assess existing or potential facilitators and barriers to successful implementation and has previously been used to investigate implementation of innovative practice in mental health care delivery (Burn et al., 2020). The focus groups were recorded and transcribed verbatim. Transcripts were coded deductively based on the five core CFIR framework domains, following which additional codes were developed inductively (Braun & Clarke, 2006; Clarke & Braun, 2013). All transcripts had a minimum of two independent coders to facilitate investigator triangulation, all of whom had prolonged engagement with the subject matter. Following independent coding, the research team met via Microsoft Teams and discussed, agreed and named themes through use of virtual post‐it notes and conceptualised the presentation of these to accurately reflect the data. This team approach to analysis can instil confidence in the findings of the evaluation.

### 2.1 Ethics

Each individual participant of the focus groups was provided with an information sheet and provided written consent for participation, video‐audio recording, and publication of the findings. The study was registered as a service evaluation, with an implementation process focus, within the research and development department of Norfolk and Suffolk NHS Foundation Trust (2022MH16‐SE), due to meeting stipulations relevant to service evaluation rather than audit or research (Moule et al., 2016). Each participating service provided confirmation as to the requirement of service evaluation approval within their own organisation and approval was gained as necessary. Each service consented to be named as a participating service, however it was agreed that individual quotes would be anonymised. Therefore each transcript was allocated a number and any identifying information removed to protect anonymity of individual quotes.

## 3 RESULTS

**FIGURE 1:**
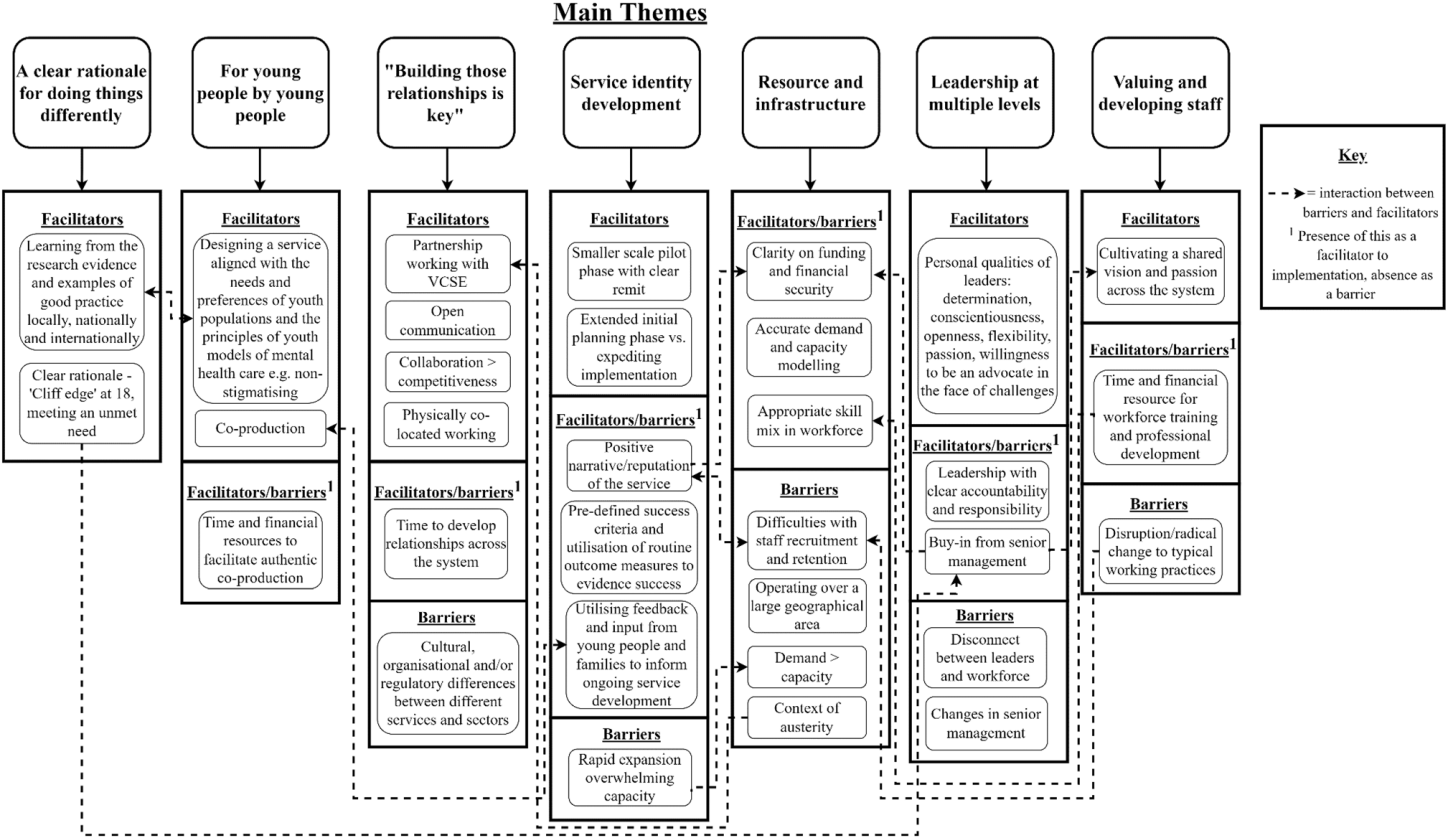
Diagram of facilitators and barriers within the main themes.

Seven themes were identified as reflecting barriers and facilitators in the process of implementing youth models of mental health care (see Figure 1 for a schematic diagram of the main themes). These themes were mapped to the main CFIR constructs and sub‐constructs (see Table 1).

**TABLE 1:** Main themes mapped onto relevant CIFR constructs and sub‐constructs.

Main theme	Overarching CFIR construct	CFIR sub‐constructs
A clear rationale for doing things differently	Intervention characteristics	Evidence strength & quality Complexity Design quality & packaging
	Outer setting	Patient needs & resources External policy & incentives
	Inner setting	Implementation climate (tension for change, relative priority)
For young people by young people	Intervention characteristics	Adaptability
	Outer setting	Patient needs & resources
	Process	Planning
Building those relationships is key	Outer setting	Cosmopolitanism
	Inner setting	Networks & communications Culture
	Process	Engaging (external change agents)
Service identity development	Intervention characteristics	Trialability Adaptability
	Inner setting	Implementation climate (goals & feedback, learning climate)
	Process	Planning Reflecting & evaluating
Resource and infrastructure	Intervention characteristics	Cost
	Inner setting	Structural characteristics Readiness for implementation (available resources)
Leadership at multiple levels	Inner setting	Networks & communication Implementation climate (learning climate) Readiness for implementation (leadership engagement)
	Characteristics of individuals	Other personal attributes
	Process	Engaging (opinion leaders, formally appointed internal implementation leaders, champions)
Valuing and developing staff	Inner setting	Networks & communications Implementation climate (compatibility, relative priority) Readiness for implementation (available resources)
	Characteristics of individuals	Knowledge & beliefs about the intervention Individual stage of change Individual identification with organisation
	Process	Reflecting & evaluating

Abbreviations: CFIR, consolidated framework for implementation research.

### 3.1 A clear rationale for doing things differently

Services reflected that the implementation process required radical change within their organisation, and it was important to have a clear rationale to justify this:

It's absolutely essential to make the case for change, make it really clear as to why there should be change. (Service 4)

Services described a sense that current models of mental health care were not meeting the needs of youth populations, using the term *“cliff edge”* to describe a drop‐off in young people accessing appropriate mental health care once above the age threshold for CAMHS. One service described how a serious incident had *“focused hearts and minds”* (Service 3). Services commented that it was often clinicians leading initial discussions around implementing change in service provision.

Research evidencing positive outcomes and cost‐savings of early intervention models of mental health care also formed a key component of services' rationale for change. Services commented that they had drawn inspiration from examples of good practice within their organisation and at a national level. The longest‐established sites reflected that there had been a reciprocal relationship whereby national policy had shaped implementation, and innovative practice within their services subsequently shaped national policy. Services described consulting global literature on the implementation of youth models, particularly from Australia:

One of the big academic bits of support for the model was TRACK study … also a lot of Australian research and other national research, particularly the early intervention psychosis evidence that had been building. (Service 6)

### 3.2 For young people by young people

Alongside research and clinical observations, services placed great emphasis on the perspectives of young people themselves and using their insights to develop services responsive to their preferences and developmental needs:

I bang that drum all the time, ask young people what they want rather than diagnose what they need. (Service 1)

Services engaged in co‐production with young people and their families, including involving young people in designing, naming and selecting the physical location of services; participation in staff interview panels; and development of staff training. Services reflected on potential challenges of co‐production, including navigating different perspectives and the need for dedicated time and financial resource to ensure authenticity,

It's the least tick‐box co‐production I've ever seen, young people really do have a voice … we think we know what we want to be doing … and young people want to do other things … working out the way forward is fascinating and exciting. (Service 3)

Services commented on key principles of youth‐focused service models. This entailed providing holistic support for a range of psychosocial needs in a non‐stigmatising environment which was physically accessible to young people in their communities:

The model provides a whole range of services all under one roof. It ensures that they are accessible, they are non‐stigmatising and that there's multiple entry routes. (Service 5)

### 3.3 Relationship building is key

Services described statutory and voluntary, community and social enterprise (VCSE) providers working together to provide a holistic service for young people, highlighting strong connections within local communities as a key benefit of VCSE providers. Building mutually beneficial relationships between providers, where all partners felt equally valued, was seen as essential to successfully implementing and sustaining services. Differences between providers, such as working hours, IT systems, levels of resource and governance practices, were described as potential barriers to forming effective relationships and working practices:

What we tried to do was fit the [VCSE] into an NHS model rather than seeing them for the strengths they had … we burdened some of those smaller [VCSE] organisations with our NHS processes. (Service 6)

Intra‐organisational cultural differences between services, most notably CAMHS and adult mental health services, were also seen as a barrier to forming good relationships across systems. For example, services reflected that CAMHS, and adult mental health services often took different approaches to understanding mental health difficulties and used different language, which could sometimes lead to differences in opinion between clinicians and tension between services:

CAMHS are trying to not label people [who are] still developing and emerging, whereas in adult services, diagnostic criteria is their gateway… so there's this huge culture gap. (Service 3)

Dedicating adequate time to the initial development of relationships across the system, emphasising collaboration over competitiveness, co‐located working practices, and creating opportunities for regular, open communication between different services were seen as beneficial to overcoming these inter‐ and intra‐organisational cultural differences:

Difficult conversations are what make healthy relationships. (Service 5)

### 3.4 Service identity development

There were differing opinions between services as to whether it was more beneficial to dedicate substantial time to planning and developing the service model prior to implementation or, conversely, to expedite the implementation phase and adapt and develop the service model as necessary. This reflected the differing organisational contexts of services, with some services reflecting on the need to act opportunistically:

When we set out, we were kind of fumbling in the dark a little bit and just working it out as we went along. (Service 2)

Services described the development of a positive, or potentially negative, ‘narrative’ or reputation of the service over time with related consequences for funding, recruitment, and support for the service model across the system:

Having a high profile really, really helps because then when a new partner comes in or a new CEO, it's good that the project has a very good national profile. (Service 3)

Services that defined success criteria pre‐implementation and routinely collected outcome measures to evidence service performance reflected that this was beneficial in terms of generating support within their organisation. Additionally, feedback from young people and families was seen as helpful to shaping ongoing service development. Services that collected fewer standardised outcome measures recognised that this could potentially represent a barrier to continued development and sustainment of their service.

I asked people how do we know that we're any good? […] where's the evidence? How do we know that we are? And I think that right from the beginning gives you some strength. (Service 1)

A number of services described successful pilot phases, during which the remit of the service was clear and smaller in scale. These services reflected that premature, rapid expansion had the potential to overwhelm the capacity of a service, sometimes leading to a disconnect between initial expectations and the reality of what the service could achieve.

### 3.5 Resource and infrastructure

Services reported that financial security, from commissioners and ring‐fenced funding for the service, was integral to successful implementation and sustainment:

It needs that buy‐in at commissioner level if anything like this is gonna remotely stand a chance of working. (Service 3)

Services discussed the financial impact of the wider societal context, for example, the detrimental impact of austerity on VCSE providers at the time of implementation, and the challenges this created in terms of partnership‐working across the local system and the subsequent capacity of the service.

Services commented on the importance of ensuring that there is an appropriate skill‐mix in the workforce to work across the age range of youth and young adults. However, services felt that it was difficult to recruit and retain staff, which was a barrier to implementation and sustainment:

It's people who are our biggest challenge for resource, staff. (Service 4)

Securing sufficient resources for the local service context was seen as a key implementation facilitator; both in terms of workforce and practical infrastructure. Services described covering a large geographical area and the need for ample resources to enable the implementation of the service model across several locations in the area. One service highlighted that frequent travel across a large geographical area hampered their capacity. Services commented on the importance of accurate demand and capacity modelling when developing a service, reflecting that when demand overwhelmed capacity, this stifled their ability to adapt and innovate:

There was no capacity or ability to be creative and innovative because it was just firefighting from day one. (Service 6)

### 3.6 Leadership at multiple levels

All services commented that leadership with clear accountability and responsibility was an important facilitator of implementation or described lack of clarity in leadership as a barrier. This was relevant both at service level of initial implementation leaders and at organisational level of senior management. Leaders within senior management were seen as having the power and influence to drive implementation forwards and generate widespread support within the rest of the system:

Having buy‐in from a very senior level so that you're both top‐down and bottom‐up and you have the muscle to implement things. (Service 3)

Determination, conscientiousness, openness, flexibility, passion and willingness to be an advocate in the face of challenges were identified as key personal qualities of good leaders:

They took some very tough decisions and some very brave decisions, so I think that they were an advocate and a bit of a champion. (Service 6)

Services highlighted that a disconnect between leaders and the workforce had the potential to lead to tension and friction, potentially hampering implementation efforts. Changes in senior management, either within an organisation or in external providers, were described as potentially disruptive to implementation:

When there were management changes… they didn't always understand the agreements that had been put in place by previous management. (Service 4)

### 3.7 Valuing and developing staff

Services commented on the potential for new models of mental health care to substantially disrupt traditional working practices, and the importance of *“how you take people on this journey with you”*. It was recognised that this could be negatively experienced by staff and detrimental to workforce retention, which was a barrier to implementation and sustainment:

One of the things that I struggled with, and I certainly didn't see, was how are we supporting staff groups? … there was those that could survive, those that didn't … so there's something about how the workforce goes on that journey. (Service 6)

Services commented on training and professional development as important for valuing staff, in addition to developing competencies and skill‐mix across the workforce. It was recognised that the availability of time and financial resources could be a barrier and that this necessitated support from senior individuals:

I feel really lucky that we've been able to go on the training … there isn't a whole load of support from senior management in the Trust. I think they like the idea, but again, it's the funding. (Service 2)

Services reflected that cultivating an ongoing shared vision for young people's mental health care provision was important in terms of staff retention and stimulating new recruitment to the service. Additionally, services cited a shared passion for young people's mental health as a unifying process, on an individual and systems level, which provided the motivation to overcome barriers and challenges associated with implementation and sustainment:

Helping young people see potential they didn't know they had and facilitating and realising that… that's why we get out of bed. (Service 6)

## 4 DISCUSSION

This service evaluation explored the barriers and facilitators to implementing youth models of mental health care in England. There were often interactions between facilitators and barriers, with sequential effects for other implementation factors. For example, a clear rationale for change, which encompassed the views of young people themselves, was effective in eliciting buy‐in from senior management, with subsequent benefits for funding and available resources. Many barriers and facilitators could be conceptualised as the inverse of one another that is, leadership with clear accountability and responsibility as a facilitator versus lack of clarity around accountability and responsibility of leaders as a barrier (see Figure 1). It is important to consider the different local contexts of each service. The differing geographical, political, and cultural contexts surrounding each service, likely impacted on what was experienced as a barrier or facilitator. For example, one service reflected on the challenges of implementing their service within the context of austerity and a number of services highlighted difficulties securing sufficient resources to operate over a large geographical area.

The current findings are congruent with previous research investigating implementation of innovative practice in mental health care delivery, which identified clarity in funding, and effective leadership at multiple levels (including buy‐in from senior management) as key facilitators, and high levels of staff turnover as a key barrier to the process of implementation (Burn et al., 2020). The results presented here additionally align with literature on youth models of mental health care which have identified co‐production (Collins et al., 2017; Stubbing & Gibson, 2021), drawing upon research evidence (McGorry et al., 2022) and appropriate skill‐mix within the workforce (O'Reilly et al., 2022) as key factors underpinning successful implementation. Additionally, analysis was informed by the CFIR framework, and the findings interpreted utilising the overarching constructs and sub‐constructs (see Table 1).

As a service evaluation, the findings are limited to the specific services that participated, all of which were operating within the mental health care landscape of England. However, it is interesting to note the alignment of the findings with the global literature pertaining to the implementation of youth models of mental health care. Future implementation research on youth models may wish to consider whether some of the barriers and facilitators are common to services and systems internationally, and whether some are more specific to a UK context.

The current study was only able to represent the perspectives of individuals who chose to and were able to participate in the focus groups. Therefore, there may have been additional perspectives within organisations that differed from these viewpoints. Recruitment may have been impacted by the short time frame in which the evaluation was conducted. However, there was good representation of different professional roles across participants. A strength was the inclusion of established services, many of which had been operating for several years. This facilitated an understanding of barriers and facilitators not only to the implementation of youth services, but also for sustainment of these services.

The themes from this service evaluation were shared with participating services and further disseminated through a national webinar exploring youth models of mental health care. There was feedback from participating services that partaking in this service evaluation acted as a reflective exercise for them to identify facilitators and barriers experienced in the implementation stages and also considers how these might impact ongoing sustainment of their established service. For example, a number of services reflected that one of the biggest challenges to ongoing sustainment of their service was a discrepancy between the growing level of demand and available capacity. Several services reflected on the need for continued work around embedding outcome measures more consistently to support with ongoing evaluation, as services reflected that this was integral to maintaining support from commissioners and organisational leadership.

In conclusion, participants suggested that successful implementation of their service model required effective communication and leadership, co‐production and cross‐system collaboration. The findings also suggest that producing a service plan with shared goals and a clear vision could be beneficial to implementation. The intention is that the data presented here will offer a useful source of information for services and systems considering the implementation of similar models. The findings can assist services and systems in identifying potential challenges they may face in their implementation journey, so that they may plan in advance how to circumnavigate these barriers. It is hoped that this will facilitate a greater chance of successful implementation and sustainment of youth models of mental health care, and thus hopefully greater national parity in mental health care for youth populations.

## FUNDING INFORMATION

This study received no specific grant from any funding agency in the public, commercial, or not‐for‐profit sectors.

## CONFLICT OF INTEREST STATEMENT

The authors declare no conflict of interest.

## Supporting information


**Table S1.** Information on participating services.

## Data Availability

The data that support the findings of this study are available from the corresponding author upon reasonable request.
