# Supplementary material for: Barriers and facilitators in the implementation of youth and young adult models of mental health care
Source: Early Interv Psychiatry. 2024 May 20;19(1):e13555. doi: 10.1111/eip.13555 (PMC11730351; doi:10.1111/eip.13555)
Supplement: Supplementary file 1 — Table S1. Information on participating services. [file EIP-19-0-s001.docx]

**Supplementary Materials**

| **Supplementary Table 1**  *Information on Participating Services* | | |
| --- | --- | --- |
| **Service** | **Est.** | **Brief description** |
| Norfolk Youth Service | 2012 | Norfolk Youth Service is an NHS community service offering interventions to young people aged 14-25 with moderate to severe mental health difficulties. There is an emphasis on functional recovery. The service works alongside local partner agencies including VCSE organisations, education, housing and social care. |
| Forward Thinking Birmingham | 2015 | Forward Thinking Birmingham is a community and inpatient mental health partnership offering mental health assessment and treatment for young people aged 0-25. The lead provider of the service is an NHS Foundation Trust. The service works closely with a range of NHS organisations and VCSE organisations. |
| Young Person’s Advisory Service (YPAS) Liverpool | 1996 | Young Person’s Advisory Service (YPAS) is a Youth Information Advice and Counselling Service (YIACS) and a VCSE organisation that supports children and young people aged 5-25 to address their mental health and emotional well-being difficulties. The service works jointly with NHS providers, education and social care. The service operates across three locality hubs in Liverpool. |
| Mind the Gap Camden | 2014 | Mind the Gap Camden is delivered by a partnership between the local council, Clinical Commissioning Group (now Integrated Care Board) and VCSE sector. The service support young people aged 16-24 and aims to improve the experience and outcomes of transitions between CAMHS and AMHS services. Elements of the service include: a transitions champion post, transition protocol, bi-weekly transitions meeting, The Hive (a locality hub setting offering a range of interventions), and the Brandon Centre (a VCSE organisation offering counselling and psychotherapy). |
| i-Rock East Sussex | 2016 | i-Rock East Sussex is a service for young people aged 14-25, co-funded by NHS and local authority, which offers advice and support for emotional and mental wellbeing, employment, education and housing. The service works in partnership with other NHS services and VCSE organisations. The service operates across three drop-in sites across East Sussex. The iRock model is in the process of being expanded across the rest of Sussex, including Brighton and West Sussex. The model maintains ongoing co-production with young people and is considering rebranding, linking closer with the Headspace movement internationally. |
| Connect 18 Somerset | 2018 | Connect18 in Somerset is an NHS community service offering intensive outreach support to young people aged 18-20 with complex or severe mental health difficulties. They offer a phased care model (up to three phases over an 18 month period ) and provide clinical interventions as well as practical support for young people and their families / carers. Each young person is allocated a support worker to “walk alongside their journey” as well as a key worker to develop holistic personalised plans and a focus on psycho education, formulation and stabilisation (phase 1), specific interventions around anxiety, depression, mentalisation based treatment MBT (phase 2) and attachment to life (phase 3). |

*Note:* AMHS=Adult mental health service; CAMHS = Child and adolescent mental health service; Est.=Established; NHS=National Health Service; VCSE=Voluntary, community and social enterprise.

**Interview guide**

| **Introductory questions** | |
| --- | --- |
| CFIR construct  N/A  N/A  Adaptability, Patient Needs & Resources  Cost | **Can you briefly summarise how your youth/young adult MH service model/initiative operates?**   - *What is the specific age range?* - *What are the referral criteria?* - *What interventions are offered?*   **At what stage of implementation is the youth/young adult MH service model/initiative at in your organization?**   - *How long has it been established/operational for?*   **Was the service model/initiative developed in co-production with young people & parents/carers?**   - *[If yes] Can you briefly describe what this involved and how this influenced the development of the service/initiative?* - *[If no] what were the barriers to this?*   **Can you briefly describe the funding/commissioning arrangements?**   - *Who/how were these funded?* - *Were any changes in funding/investment necessary?* - *Was/is demand capacity modelling used to consider the impact of a service change?* |
| CFIR construct  Intervention source, Planning, Engaging  External Policies & Incentives  Relative Advantage  Planning, Engaging (Opinion Leaders, Formally Appointed Internal Implementation Leaders), Other Personal Attributes  Cosmopolitanism  Networks & Communications, Implementation Climate (Compatibility)  Networks & Communications,  Engaging (Key Stakeholders, Intervention Participants) | **Who was involved in initial conversations or meetings about introducing a youth/young adult MH service model/initiative in your organisation(s)/system?**   - *Who was responsible/accountable for decision making? (‘pressing go’)*   **What were the motivating factors behind introducing this service/initiative in your organisation?**  **-**Was the model/initiative informed by any research/evidence?  **What were the anticipated benefits of introducing this service model/initiative?**  **Who was key to the planning and implementation phase of the service model/initiative?**   - **Were there specific roles responsible and accountable for implementation?** - *What were their roles? Where were they situated?* - *Who led on the implementation of the service model/initiative itself?* - *How did this person/people come into this role?* - *What attributes or qualities were important to this/these implementation role?* - *Did commissioners remain involved during the implementation phase?*   **Did you work with external agencies and networks across your system to support the implementation of the youth/young adult service model/initiative?**   - *[If yes] how did this influence the development and implementation of the service model/initiative?* - *What was their level of involvement?*   **How did you work across child and adult services to implement the service model/initiative?**   - *What helped that way of working/helped people to communicate and work together during the early implementation phase?* - *Is there anything that could have been done differently that could have helped make this better?*   **How did you engage with wider stakeholders/organisations/systems to support with implementation?**  **How did you engage with staff across services to support with implementation?**  **How did you engage with servicer users and / or parents/carers to support with implementation?**  **What was the general level of receptivity within the organisation(s)/system to introducing the service model/initiative?**   - *Why do you think this was?*   **Do you think the wider organisational/system culture(s) affected the implementation of the service?**   - *[If yes] how?*   **What kind of support or actions were taken by leaders in your organisation(s)/systems to assist with implementation?**   - *Was there a sense that leaders were taking accountability for implementation?*   **What changes were necessary in terms of the structure of the organisation(s)/systems to deliver this new service model/initiative?**   - *What resources were needed? (Human resource, IT)* - *Are there any further resources still needed?* - *If changes were based on research findings, how easy were these to apply in your context?*   **During the planning phase of your service model/initiative, what challenges to implementation were predicted to arise?**   - *Did any additional unpredicted issues or complications arise during the implementation phase?* - *How did you overcome these?*   **Was there any kind of pilot project/proof of concept of the service model/initiative prior to full-scale implementation?**   - *[If yes] can you briefly summarise what this involved?* - *What was the learning form these initial pilots?*   **Were there set goals/success criteria in relation to the introduction/implementation of the service model/initiative?**   - *[If yes] Who was involved in the generation of these goals/criteria? Where did they come from?* - *what were the goals? How were these monitored for progress?*   **What kind of information was collected to evaluate the implementation of the service model/initiative?**   - *How was this collected?* - *Was feedback elicited from staff?* - *Was feedback elicited from service users?* - *[If yes] what kind of feedback?* - *How was this information used?* - *Has ongoing evaluation and feedback been embedded into the service?* |
| Implementation Climate  Culture  Readiness for Implementation (Leadership Engagement)  Structural Characteristics, Readiness for Implementation (Available Resources)  Planning, Implementation Climate (Learning Climate)  Trialability  Implementation Climate (Goals and Feedback)  Reflecting & Evaluating |  |
| **Closing questions** | |
| Knowledge & Beliefs about the Intervention | **In terms of what you had initially planned/hoped for in terms of the implementation of your service model/initiative, would you say the current position and functioning of your service model/initiative is aligned with what was hoped for?**   - *[If no] why do you think this is?* - *Is this model now embedded as part of the local Mental health landscape?* - *Do you continue to use service user feedback to shape service development?*   **Is there anything else you’d like to comment on regarding key facilitators or barriers to implementation that we haven’t covered so far?**  **Knowing what you know now, what advice would you give to other organisations/systems thinking about embarking the implementation of a youth/young adult MH service model or initiative?** |
